# Gemcitabine treatment induces endoplasmic reticular (ER) stress and subsequently upregulates urokinase plasminogen activator (uPA) to block mitochondrial-dependent apoptosis in Panc-1 cancer stem-like cells (CSCs)

**DOI:** 10.1371/journal.pone.0184110

**Published:** 2017-08-30

**Authors:** Li Wang, Yi Zhang, Weiguo Wang, Yunjie Zhu, Yang Chen, Bole Tian

**Affiliations:** Department of Pancreatic Surgery, West China Hospital, Sichuan University, Chengdu, P.R. China; University of South Alabama Mitchell Cancer Institute, UNITED STATES

## Abstract

**Background:**

Pancreatic ductal adenocarcinoma (PDAC) is an aggressive cancer with poor survival rates. The presence of cancer stem-like cells (CSCs) is believed to be among the underlying reasons for the aggressiveness of PDAC, which contributes to chemoresistance and recurrence. However, the mechanisms that induce chemoresistance and inhibit apoptosis remain largely unknown.

**Methods:**

We used serum-free medium to enrich CSCs from panc-1 human pancreatic cancer cells and performed sphere formation testing, flow cytometry, quantitative reverse transcription polymerase chain reaction (RT-qPCR) and semi-quantitative western blotting to confirm the stemness of panc-1 CSCs. Hallmarks of endoplasmic reticulum (ER) stress, including IRE1, PERK, ATF4, ATF6α, GRP78 and uPA expression, were detected after gemcitabine treatment. Effects of gemcitabine-induced uPA expression on cell invasion, sphere formation, colony formation and gemcitabine sensitivity were detected. Electrophoretic mobility shift assays (EMSAs) and RNA-immunoprecipitation (RIP) were performed to detect interaction between the uPA mRNA 3’-UTR and mutant p53-R273H expressed by panc-1 CSCs. The effects of upregulated uPA by gemcitabine on apoptosis were detected by Annexin V-FITC/PI staining, and the impact of uPA on small molecule CP-31398-restored mutant p53 transcriptional activity was measured by a luciferase reporter assay.

**Results:**

Enriched panc-1 CSCs expressing high levels of CD44 and CD133 also produced significantly higher amounts of Oct4 and Nanog. Compared with panc-1 cells, panc-1 CSCs presented chemoresistance to gemcitabine. ER stress gene detections demonstrated effects of gemcitabine-induced ER stress on both the pro-apoptotic and pro-survival branches. ER stress-induced ATF6α upregulated level of uPA by transcriptionally activating GRP78. Gemcitabine-induced uPA promoted invasion, sphere formation and colony formation and attenuated apoptosis induced by gemcitabine in panc-1 CSCs, depending on interaction with mutant p53-R273H. Upregulation of uPA abolished CP-31398-mediated restoration of mutant p53 transcriptional activity in panc-1 CSCs.

**Conclusion:**

Gemcitabine treatment induced ER stress and promoted mutant p53-R273H stabilization via transcriptionally activated uPA which may contribute to chemoresistance to gemcitabine. Notably, upregulation of uPA by gemcitabine treatment may lead to the failure of CP-31398; thus, a novel strategy for modulating mutant p53 function needs to be developed.

## Introduction

Pancreatic ductal adenocarcinoma (PDAC) is the fourth leading cause of cancer-related death in the United States and is one of the most aggressive and lethal malignancies [1]. Despite much focus on the molecular mechanisms of carcinogenesis, chemoresistance and recurrence, the prognosis of PDAC remains poor. Based on emerging evidence demonstrating the presence of a small subset of cells, termed cancer stem-like cells (CSCs), these cells are considered a sub-population that contributes to tumour growth, chemoresistance, invasion, metastasis and recurrence [2].

Gemcitabine, a cytotoxic nucleoside analogue and one of the most widely used chemoagents in pancreatic cancer [3], frequently induces chemoresistance upon long-term use [4]. To avoid such chemoresistance, induction of an organelle-related stress response, such as the endoplasmic reticulum (ER) stress response in pancreatic cancer cells, has proved to be promising [4]. Indeed, ER stress results in activation of the unfolded protein response (UPR), which stimulates the pro-survival branch via up-regulation of protein chaperones and ER-associated protein degradation (ERAD). According to literature, UPR signaling pathways contain three arms to promote cell survival or death, namely inositol requiring enzyme 1 (IRE1), double-stranded RNA-activated protein kinase like ER kinase (PERK), and activating transcription factor 6 (ATF6) [5]. Although it is unclear how the ER-UPR pathways control the balance between life and death, the kinetics of IRE1, ATF6 and PERK activation and inactivation regulate both pro-survival and pro-apoptotic branches [6]. Early activation of the ER-UPR triggers pro-survival signal pathways, including folding chaperones to promote protein folding in cells. Chronic activation of this pathway is found to generate pro-apoptotic signals through the PERK signal pathway to induce cell death [6–8]. Cheng and colleagues showed that induction of ER stress in panc-1 pancreatic cancer cells by Pachymic acid inhibits cell growth and induces apoptosis [9], and further evidence demonstrates that chemically trigged ER stress such as that caused by 1,1-bis(3'-indoly)-1-(p-substituted phenyl) methane [10], 3,3'-diindolylmethane [11] and Bortezomib, induces apoptosis in panc-1 cells [12]. However, less is known about the exact effect of ER stress on CSCs, including panc-1 CSCs. It has been reported that tunicamycin-induced ER stress promotes apoptosis in a monolayer of cervical cancer cells but that this was not observed after sphere-formiation [13]. These findings indicate the potential of ER stress to induce the opposite effects on monolayer and sphere-forming cells.

Urokinase plasminogen activator (uPA) is tightly correlated with increased epithelial-mesenchymal transition (EMT), which contributes to a high rate of recurrence of pancreatic cancer [14, 15]. Khanna and colleagues revealed that a direct regulatory effect of uPA expression was desensitization of pancreatic cancer cells to gemcitabine-induced apoptosis [16]. Furthermore, high levels of uPA and its receptor (uPAR) indicate a high frequency of relapse after primary therapy, high possibility of recurrence and metastasis, and poor prognosis [17, 18]. It is reported that the 3’ untranslated region (UTR) of the uPA mRNA binds specifically to the C-terminus of the p53 protein (364–393 aa) and thus disrupts p53’s regulatory roles function towards certain downstream target genes, without interfering with p53’s DNA binding activity or promoter transactivation [19]. It has also been revealed that RNA binding may compete with p53 C-terminal acetylation, suggesting possible effects of RNA binding on p53’s transcriptional activity towards its downstream target genes [20]. Accordingly, much controversy remains.

CP-31398 [N′-[2-[2-(4-methoxyphenyl)ethenyl]-4-quinazolinyl]-N,N-dimethyl-1,3-propanediamine dihydrochloride] is a synthetic styrylquinazoline that can restore a wild-type-associated epitope to the DNA-binding site of the mutant p53 protein [21]. The effects of such restorationcan induce growth arrest or apoptosis in human cancer cells harbouring mutant p53, including in panc-1 cells carrying a mutation in codon 273, R273H (Arg to His), which abolishes its ability to transactivate downstream target genes [22]. Furthermore, CP-31398 also causes marked post-transcriptional upregulation of p53 protein levels and promotes the activity of wild-type p53, leading to cell cycle arrest and promoting apoptosis by blocking its ubiquitination and degradation [23]. However, the effects of uPA on CP-31398-mediated restoration of p53 activity is unknown.

Here, we investigate the chemosensitivity of panc-1 CSCs to gemcitabine and ER stress due to gemcitabine treatment. Opposite the panc-1 monolayer, gemcitabine treatment induced ER stress and led to upregulation of uPA, which post-transcriptionally modulates mutant p53 R273H stability. Upregulated uPA promoted invasion, sphere formation and colony formation in vitro via stabilization of mutant p53-R273H. Furthermore, we confirmed that the effects of CP-31398 on transcriptional activation of mutant p53-R273 were abolished by gemcitabine-induced uPA. These results indicate that ER stress induced by gemcitabine treatment upregulats uPA and results in the promotion of malignant behaviours. CP-31398, as a strategy for restoring mutant p53, partially fails to inhibit growth and invasiveness of panc-1 CSCs carrying mutant p53.

## Material and methods

### Cell culture

Human pancreatic cancer cell panc-1 was obtained from American Type Culture Collection (ATCC Manassas, VA, USA) and cultured in Dulbecco Modified Eagle Medium (DMEM, Gibco, Paisley, UK), supplemented with 10% fetal bovine serum (FBS, Gibco, Paisley, UK), 100 U/ml pencillin and 100 U/ml streptomycin at 37°C incubator with 5% CO2. For isolating CSCs sub-population from Panc-1 cells, Panc-1 cells were maintained in DMEM/F12 without FBS, supplemented with 2% B27 supplement (Life Technologies, Grand Island, NY, USA), 20 ng/ml human EGF (PeproTech, Rocky Hill, NJ, USA.), 40 ng/ml bFGF (PeproTech, Rocky Hill, NJ, USA.) and 5ug/ml insulin (PeproTech, Rocky Hill, NJ, USA.). Under this culture condition, non-adherent cell aggregates referred to as spheres were formed. The medium was half-refreshed every 2–3 days. 10–14 days is required to form obvious spheres.

### Flow cytometry analysis of CD24 and CD44 expression

Panc-1 and Panc-1 CSCs were enzymatically dissociated and directly subjected to immunofluorescence staining followed by flow cytometry analysis. Briefly, the cells were washed three times with ice-cold phosphate buffered saline (PBS) and suspended in PBS supplemented with 0.5% BSA. Then FITC-isotype control (Cat.: 553478, BD Pharmingen, Franklin Lakes, NJ, USA), FITC-CD24 (Cat.: 560992, BD Pharmingen, Franklin Lakes, NJ, USA) and FITC-CD44 (Cat.: 555478, BD Pharmingen, Franklin Lakes, NJ, USA) were added respectively and stained for 30 min in the dark. The analysis was performed with 3 laser Navios flow cytometers (Beckman Coulter, Brea, CA, USA).

### RT-quantitative PCR (RT-qPCR)

Total RNA was extracted from cells using TRIzol reagent (Life Technologies, Grand Island, NY, USA), followed by reverse transcription (Roche, Basel, Switzerland). cDNA was used as template for 40-cycle amplification in ABI7500 (Applied Biosystems, Foster City, CA, USA). The expression of genes was normalized using β-actin mRNA as an internal standard by the comparative Ct method.

The primer pairs used were as follows; CD24 forward 5’- CTCCTACCCACGCAGATTTATTC-3’ and reverse 5’- AGAGTGAGACCACGAAGAGAC-3’; CD44 forward 5’- CTGCCGCTTTGCAGGTGTA-3’ and reverse 5’- CATTGTGGGCAAGGTGCTATT-3’; CD133 forward 5’- AGTCGGAAACTGGCAGATAGC-3’ and reverse 5’- GGTAGTGTTGTACTGGGCCAAT-3’; Oct4 forward 5’- CTGGGTTGATCCTCGGACCT-3’ and reverse 5’- CCATCGGAGTTGCTCTCCA-3’; Nanog forward 5’- TTTGTGGGCCTGAAGAAAACT-3’ and reverse 5’- AGGGCTGTCCTGAATAAGCAG-3’.

For detecting XBP1/s and XBP1, the primer pairs used were as follows; human total XBP1 (XBP1) forward 5’-GGCATCCTGGCTTGCCTCCA-3’ and reverse 5’-GCCCCCCTCAGCAGGTGTTCC-3’; human spliced XBP1 (XBP1/S) forward 5’- CTCAGACTACGTGCACCTCTGC-3’ and reverse 5’- CCAACAGGATATCAGACTCT-3’; β-actin forward 5’-CATGTACGTTGCTATCCAGGC-3’ and reverse 5’-CTCCTTAATGTCACGCACGAT-3’.

### Semi-quantitative PCR for detecting XBP1 and XBP1/s

For semiquantitative detection XBP1/s and XBP1, the primer pairs used were as follows: semi-XBP1 forward 5’- GGGGAATGAAGTGAGGCCAG-3’ and reverse 5’- TTCTGGAGGGGTGACAACTGG-3’. Amplification using PCR SuperMix High Fidelity (Life Technologies) was performed using the following cycling parameters: pre-incubation at 94°C for 5min, 35 cycles of 94°C for 10 sec, 60°C for 10 sec, 72°C for 10 sec. After amplification, PCR products were fractionated in 10% TBE-PAGE gel and stained using 0.5μg/ml ethidium bromide (EB) for 10 min.

### Fractionation of nuclear protein and cytosolic protein

For isolation of nuclear and cytosolic fractions, Panc-1 CSCs treated with or without gemcitabine were lysed by adding lysis buffer (10 mM HEPES-KOH, pH7.5, 10 mM MgCl_2_, 1mM EDTA, 0.5% NP-40, 1 mM DTT, 10uM PMSF) and incubated for 10 min at 4°C. Cells were pelleted by centrifugation for 1min at 12,000 g at 4°C. Supernatants were collected as cytosoli fraction and the pellet was resuspended in 100 ul of Nuclear-fraction lysis buffer (HEPES-KOH, pH7.5, 25% glycerol, 400 mM NaCl, 1.5 mM MgCl2, 0.2 mM EDTA, 0.5 mM DTT and 10Um PMSF). Lysates were incubated for 30 min on ice followed by being pelleted via centrifugation at 12,000g at 4°C for 5min. Supernatant was collected as nuclear fraction. All fractionated samples were measured for protein concentration using Bradford reagent and their purification was measured by determining lamin A/C and β-actin using Western blot, respectively.

### Apoptotic analyses

Annexin V-FITC/PI Apoptosis Detection Kit (Abcam, Cambridge, England) was employed. Cells with indicated treatment were collected, washed with ice-cold PBS for three times, and resuspended in staining buffer supplemented with Annexin V-FITC and PI according to the manufacturer’s instructions. The sample was analyzed by flow cytometry with 3 laser Navios flow cytometers (Beckman Coulter, Brea, CA, USA) in 30 min.

### Lentiviral shRNA knockdown

shRNA construct in the pLKO.1 lentiviral vector containing the following uPA targeting sequences (5’-AGCTGAGAGCCCTGCTGGCGCG-3’) or GRP78 targeting sequences (5’-CTTGTTGGTGGCTCGACTCGA-3’). uPA shRNA lentiviral particles (LV-uPA-KD) or GRP78 shRNA lentiviral particles (LV-GRP78-KD) was produced by co-transfection of targeting plasmids and packaging vectors (psPAX2 and pMD.2G), using lipofectamine 2000 (Life Technologies, Grand Island, NY, USA) into HEK293T cells (ATCC Manassas, VA, USA). Viral supernatants were collected by centrifugation and used for further experiments.

### Western blotting

Mouse Anti-human CD24 antibody (Cat.: ab30350), rabbit anti-human CD44 antibody (Cat.: ab157107), rabbit anti-human CD133 antibody (Cat.: ab19898), rabbit anti-human Oct4 antibody (Cat.: ab19857), rabbit anti-human Nanog antibody (Cat.: ab21624), rabbit anti-IRE1 (phosphor S724) antibody (Cat.: EPR5253), rabbit anti-IRE1 antibody (Cat.: ab96481), rabbit anti-PERK (phosphor T982) antibody (Cat.: ab192591) and rabbit anti-PERK antibody (Cat.: ab65142) were obtained from Abcam (Cambridge, England) and diluted followed the manufacturer’s instructions.

Total protein was prepared using RIPA buffer (Thermo Scientific, Waltham, MA, USA) followed the manufacturer’s instruction. The same amount of protein from total protein was fractionated using Tris-glycine gels, and transferred to PVDF membranes. After transferring, PVDF membranes were blocked in 5% milk/TBS buffer at room temperature for 30 min, and this was followed by an incubation with primary antibodies at 4°C overnight. After washing with PBS-T (containing 0.1% Tween-20) for three times, HRP-conjugated secondary antibodies were incubated with PVDF membranes for another 1 hour. Blots were developed using Pierce^™^ ECL Western Blotting Substrate (Thermo Scientific, Waltham, MA, USA) according to the manufacturer’s instructions.

### Cell proliferation and colony formation assays

CCK-8 assay was employed for determining cell proliferation. 1×10^4^ Cells were seeded to 96-well plates, and maintained in DMEM/F12 medium for 24h. After incubation with indicated concentration of gemcitabine (0.01, 0.1, 1, 2.5, 7.5, 10, 20 μM) for 24h, 10μl CCK-8 (Sigma—Aldrich, St. Louis, MO, USA) was added into each well and incubated at 37°C for 4h. The absorbance at 490 nm was measured with a microplate spectrophotometer (Synergy 2 Multi-Mode Microplate Reader; BioTek, Winooski, VT, USA).

For the colony formation assay, 1×10^3^ cells were seeded into 12-well plates in 0.3% soft agar for 14 days. Cells were maintained until visible coonies were observed. Plates were stained with 0.5% crystal violet for 30min and imaged under a X71 (U-RFL-T) fluorescence microscope (Olympus, Melville, NY).

### Invasion assays

Transwell chambers were pre-covered with 80μL Matriel diluted with DMEM/F-12 and maintained in 37°C incubator overnight. 2.5×10^3^ singled cells were suspended in 200 μL DMEM/F-12 medium and seeded in the upper chambers, and 600 μL DMEM/F-12 supplemented with 2% B27 supplement, 20 ng/ml human EGF, 40 ng/ml bFGF and 5ug/ml insulin. After incubation at 37°C, the cells were fixed with 4% paraformaldehyde for 10 min, stained with 0.5% violet crystal for 30min, and imaged under a X71 (U-RFL-T) fluorescence microscope (Olympus, Melville, NY).

### CP-31398 treatments

Cells were grown in 100-mm plates at 80% confluency and treated with 0, 1, 5, 10, 20, and 40 μg/ml of CP-31398 (Sigma-Aldrich, St. Louis, MO, USA) for 24 hours or with 20 μg/ml of CP-31398 for 0, 3, 6, 12, 18, and 24 hours. Following treatment, cells were harvested and lysed in 100 μl of RIPA lysis buffer as described below.

### Caspases 3/7 activity assay

Cells were seeded in 96-well plates at a density of 10,000 cells/well with indicated amount of gemcitabine for 24-h incubation. The supernatant was refreshed with DMEM supplemented with 10% FBS and 40 ul Caspase-Glo reagent (Promega, Madison, WI, USA) per well. 2h later, plates were read using microplate reader (Synergy 2 Multi-Mode Microplate Reader; BioTek, Winooski, VT, USA).

### Transient transfection and luciferase assays

For detecting the activation of ATF6, 5×ATF6GL3 plasmid (Addgene, Cambridge, MA, USA) containing five copies of ATF6 binding site (5’- CTCGAGACAGGTGCTGACGTGGCGATTC-3’) was transfected into Panc-1 CSCs using Lipofectamine^™^ 2000 (Life Technologies, Grand Island, NY, USA) according to the manufacturers protocol. The transfected cells were pretreated with 1.5μM GSK2606414 (a PERK inhibitor; PERKi), 7μM 4μ8C (an IRE1α inhibitor; IRE1i) or 1.87μM gemcitabine as indicated for 24h. Luciferase activity was monitored using the dual-luciferase reporter assay kit (Promega, Madison, WI, USA), according to the manufacturers protocol.

To test whether uPA silence or gemcitabine treatment is able to affect restorating effects of CP-31398 on transcriptional activity of mutant p53, luciferase reporter plasmids pG13-Luc (containing 13 tandem p53-binding elements) was transiently transfected into Panc-1 CSCs before treatment. The transfected cells were treated as indicated. Luciferase activity was monitored using the dual-luciferase reporter assay kit (Promega, Madison, WI, USA), according to the manufacturers protocol.

## Results

### Isolation and characterization of human pancreatic CSCs from the human pancreatic cancer cell line panc-1

**Fig 1:**
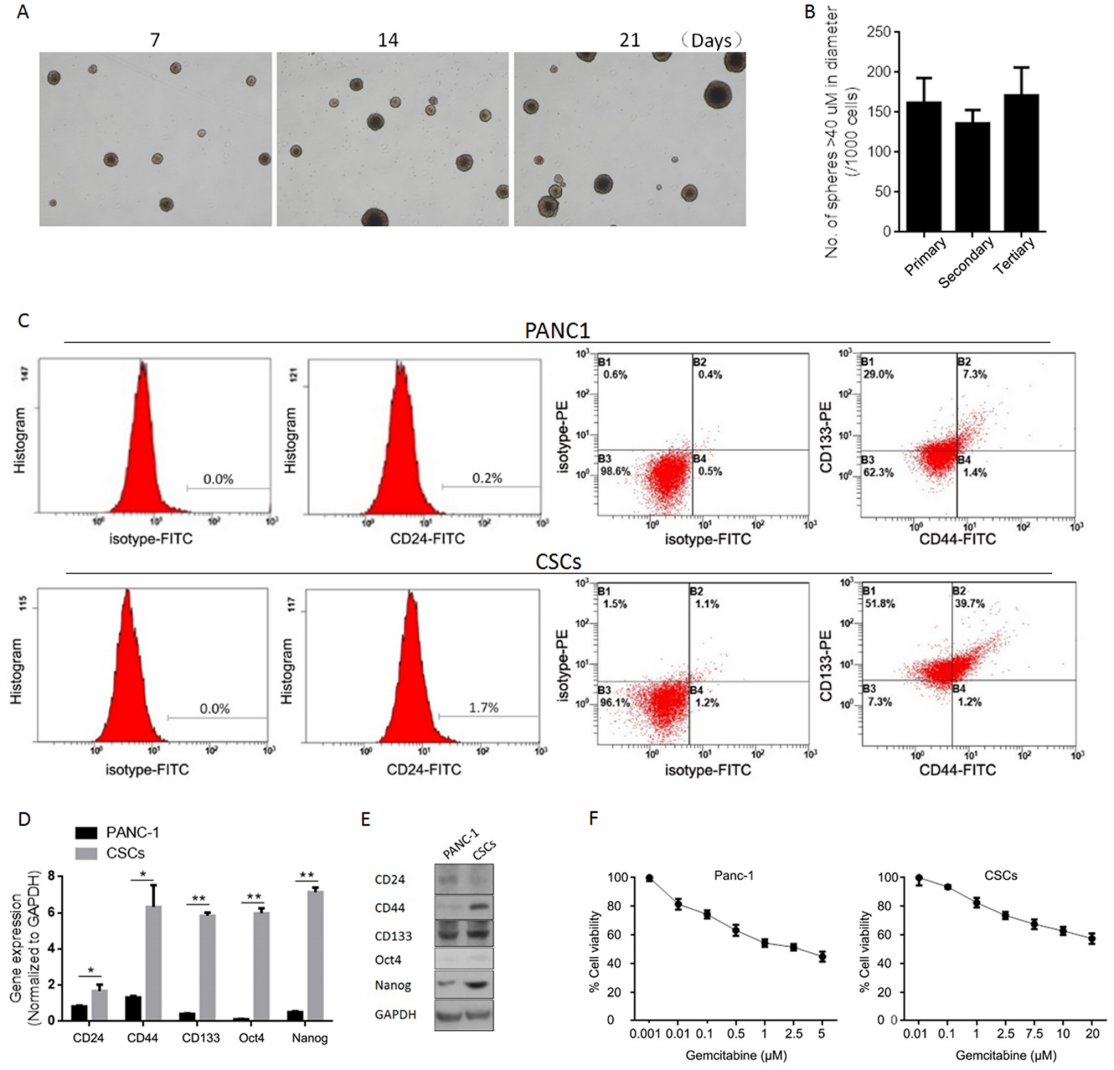
Enrichment and characterization of human pancreatic CSCs from human pancreatic cancer cell line Panc-1. (A) Sphere formation of Panc-1 CSCs at day 7, 14 and 21. (B) Self-renewal capacity of Panc-1 CSCs were tested using serial replating assay. (C) Panc-1 CSCs were analyzed by folw cytometry using antibody against CD24, CD44, and CD133. (D) Expression of stem cell markers. The expression of CD24, CD44, CD133, Oct4, and Nanog was measured by RT-qPCR. Data represent mean±SD (left panel). protein levels were assessed by semi-quantitative Western blot (righ panel). (E) Gemcitabine sensitivity of Panc-1 cells or Panc-1 CSCs were assessed. *P<0.05; **P<0.01.

The small tumor-forming population, which is characterized by its self-renewal capacity, contributes to the therapeutic failure/recurrence of chemotherapy [24], and identification and isolation of CSCs using putative surface markers contributes to modification of a therapeutic strategy. In this study, we first characterized the well-accepted cancer stem cell surface marker, including CD24, CD44 and CD133 [25, 26] in CSCs isolated by culturing panc-1 cells in serum-free medium. Spheres of panc-1 cells formed at day 7, day 14 and day 21and showed similar morphologies (Fig 1A). Serial replating assays was performed to confirm the self-renewal capacity of CSCs self-renewal capacity and it was found that panc-1 CSCs could be passaged and formed similar spheres (Fig 1B). We then identified CSC cell surface makers and found that panc-1 CSCs express CD44 and CD133 strongly and CD24 weakly (Fig 1C). To assess expression of stem cell markers (CD24, CD44 and CD133) and pluripotency maintenance factors (Oct4 and Nanog), quantitative reverse transcription polymerase chain reaction (RT-qPCR) and semi-quantitative western blotting analysis were performed. As expected, panc-1 cells were found to express very low levels of CD24, CD44, CD133, Oct4 and Nanog compared to CSCs (Fig 1D and 1E). We further detected panc-1 cell and CSC sensitivity to gemcitabine by the MTT (3-(4,5-dimethylthiazol-2-yl)-2,5-diphenyltetrazolium bromide) assay. The IC_30_ values calculated using CompuSyn software were as follows: panc-1, 0.175 uM; CSCs, 1.87 μM.

### Gemcitabine treatment affects both pro-apoptotic and pro-survival branches of ER stress-induced UPR

**Fig 2:**
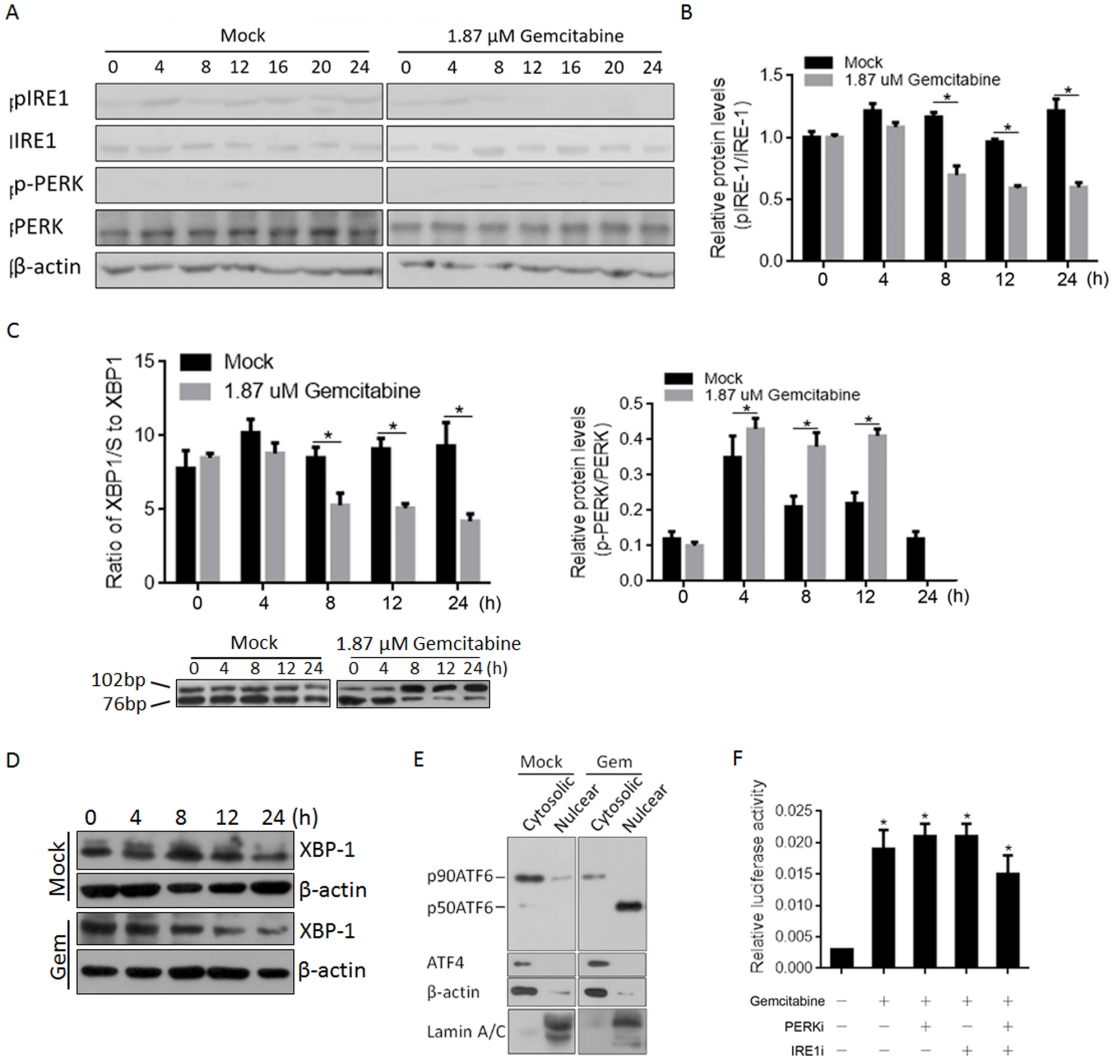
Gemcitabine treatment stimulated ER stress and activated transcriptional activity of ATF6α. (A) The expressing levels of pIRE1, IRE1, p-PERK, and PERK were measured by semi-quantitative Western blot with or without Gemcitabine treatment. (B) The respective band densitometry analyses were performed using the Image J software program. The values represent the mean±SD of three independent experiments. (C) The ratio of spliced XBP1 (XBP1/S) to XBP1 mRNA was calculated using the comparative Ct methods. (D) Protein of XBP1/S was detected by semi-quantitative Western blot. (E) The spliced form of ATF6α (p50ATF6) and unspliced form of ATF6α (p90ATF6) were detected. (F) Transcriptional activity of p50ATF6 was measured with the presence of PERK inhibitor (PERKi) or IRE1 inhibitor (IRE1i). *P<0.05.

The exhibited chemoresistance capacity of panc-1 CSCs prompted us to examine the effects of gemcitabine treatment on UPR, with a focus on pro-survival and pro-apoptotic branches in response to ER stress. Activation of three UPR sensors (IRE1, PERK and ATF6) was analysed by western blotting at several time points after treatment with 1.87 μM gemcitabine, including 0, 4, 8, 12, 16, 20 and 24 h. As shown in Fig 2A, without disturbing the total amount of IRE1 and PERK, gemcitabine treatment decreased the amount of phosphorylated IRE1 (pIRE1) and increased the amount of phosphorylated PERK (p-PERK); this attenuation of IRE1 activation and increase in p-PERK activation (Fig 2B) indicated the shift in the pro-survival branch. Unexpectedly, p-PERK rapidly decreased at 24 h after gemcitabine treatment (Fig 2B). Activated IRE1 cleaves an intron from the XBP1 mRNA, resulting in the production of a spliced-XBP1 protein [27]. To confirm activation of IRE1 by gemcitabine treatment, we assessed this spliced XBP-1 (XBP-1/s) by RT-qPCR and western blotting. As expected, XBP-1/S was decreased by gemcitabine treatment in panc-1 CSCs at both the mRNA and protein levels (Fig 2C and 2D). ER stress triggers were processed via ATF6α by decreasing its molecular mass from 90 (p90ATF6α) to 50 (p50ATF6α) kDa and inducing its translocation to nucleus. We next sought to identify the effects of gemcitabine on ATF6α in panc-1 CSCs, and a nuclear protein fraction was prepared for ATF6α detection after 24 h of treatment with or without gemcitabine. As illustrated in Fig 2E, p50ATF6α in a well-purified nuclear protein fraction was increased after gemcitabine treatment, with a slight disruption in the total p90ATF6α protein in the cytoplasm. To more directly assess the transcriptional activity of p50ATF6α, we transfected cells with an ATF6α target site-luciferase reporter (5×ATF6GL3) prior to gemcitabine treatment and found that gemcitabine treatment specifically induced ATF6α transcriptional activity (Fig 2F). Application of aPERK (PERKi) or IRE1 (IRE1i) inhibitor failed to interrupt ATF6α’s transcriptional activity. We conclude that gemcitabine treatment of panc-1 CSCs promotes the pro-survival branch and inhibits the pro-apoptotic branch of UPR. However, induction of these branches by gemcitabine treatment showed a reversible tendency (Fig 2B).

### Gemcitabine regulates uPA expression via activated GRP78 and results in the promotion of proliferation and colony formation in panc-1 CSCs

**Fig 3:**
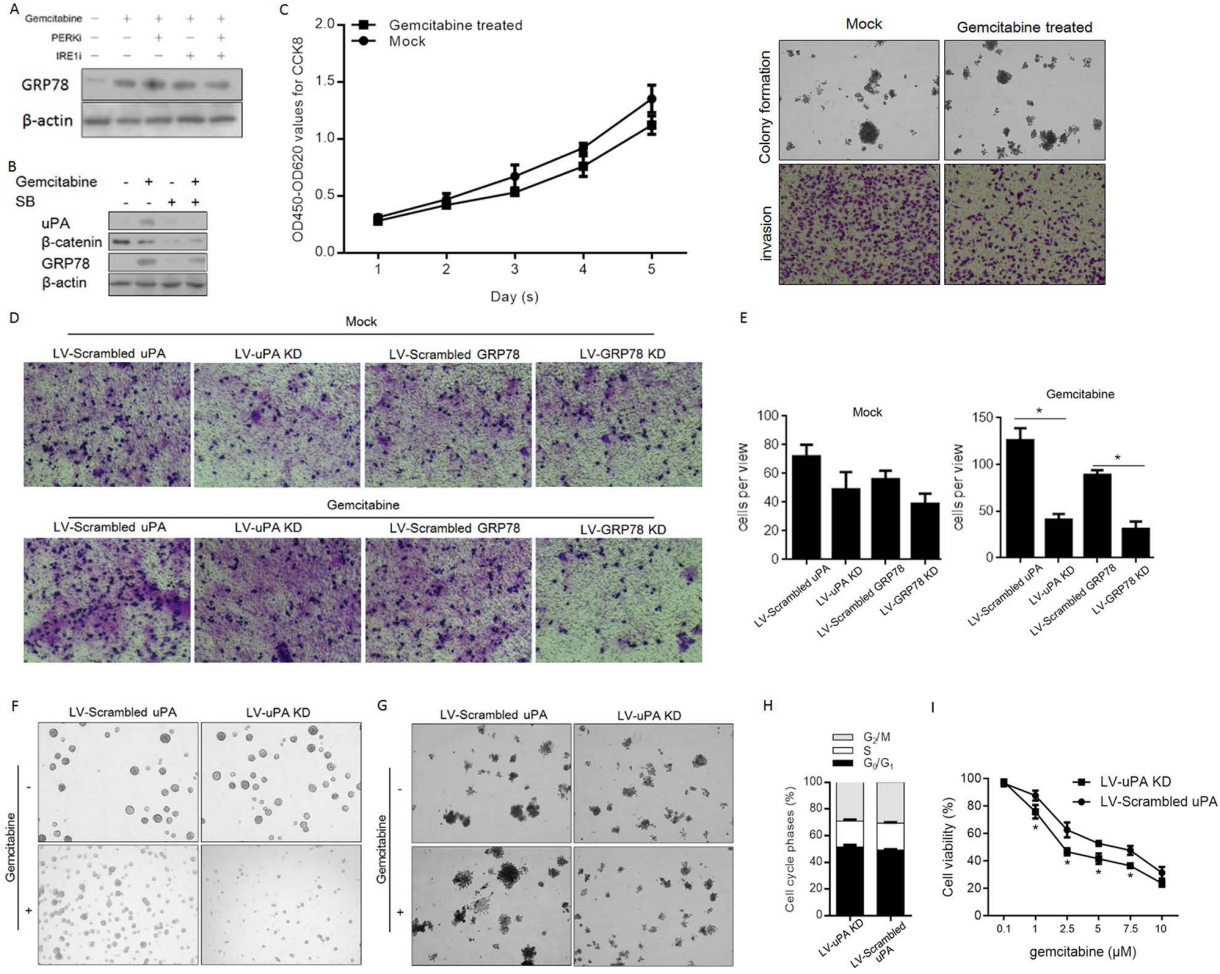
uPA expression was upregulated by Gemcitabine treatment and resulted in promotion on sphere formation, invasion, and colony formation. (A) GRP78 protein level was measured for determine whether it is modulated by gemcitabine treatment. (B) Upregulation of uPA by gemcitabine-induced GRP78. (C) and (D)Transwell assay for analyzing the effects of uPA or GRP78 on invasive capacity of Panc-1 CSCs. (E) Sphere formation assay for determining the effect of uPA. (F) Soft agar assay was performed for determining the effect of uPA on colony formation ability in Panc-1 CSCs. (G) Percentage of cell phase was detected by PI staining. (H) The effects of uPA on Gemcitabine sensitivity in Panc-1 CSCs. *P<0.05.

Active ATF6α has been indicated as a key regulator of the ER stress pathway because it can activate expression of GRP78 [28]. Thus, to confirm whether gemcitabine treatment activates ATF6α, GRP78 protein levels were detected. As shown in Fig 3A, without disturbance due to the presence of PERKi or IRE1i, gemcitabine treatment stimulated expression of GRP78. UPA expression is reported to be activated by GRP78 viaβ-catenin signaling [29], which prompted us to employ sodium butyrate (SB), a suppressor of β-catenin signaling, to confirm whether gemcitabine-mediated upregulation of GRP78 stimulates uPA expression. As expected, gemcitabine treatment upregulated both GRP78 and uPA, and addition of a β-catenin suppressor reduced uPA expression at both mRNA and protein levels, without altering GRP78 expression (Fig 3B). As uPA upregulation reportedly promotes migration and invasion of colon cancer cells [30], we detected its effects on proliferation, colony formation and invasion. Lentivirus was then employed for silencing. After confirming the silencing efficiency of LV-uPA-KD (S1A and S1B Fig) and LV-GRP78-KD (S2A and S2B Fig), panc-1 CSC proliferation, colony formation and invasion were assessed. Gemcitabine treatment markedly promoted invasion of panc1 CSCs without disturbing proliferation and colony formation (Fig 3C). Additionally, silencing of both GRP78 and uPA decreased panc1 CSC invasion (Fig 3D & 3E).

UPA is highly expressed in pancreatic CSCs [31] and potentially promotes chemoresistance to gemcitabine in pancreatic cancer cells [32]. Therefore, we further assessed its effects on the stemness of panc-1 CSCs and their chemosensitivity to gemcitabine. Through sphere formation assays, we observed that gemcitabine treatment only slightly affected the sphere-forming ability of panc-1 CSCs; transfection of LV-uPA KD significantly attenuated the sphere forming ability of these cells, which led to obvious disintegration of pancreatospheres (Fig 3F) and decreased colony formation ability on soft agar (Fig 3G). However, no detectable changes on cell cycle phases were observed after LV-uPA KD transfection (Fig 3H). To confirm whether inhibition of uPA expression sensitizes panc-1 CSCs to gemcitabine, we transfected LV-uPA KD or LV-scrambled uPA into panc-1 CSCs treated them with varying concentrations of gemcitabine (0–1000 nM) and found that uPA silencing indeed sensitized panc-1 CSCs to gemcitabine (Fig 3I).

### Induced uPA binds specifically to p53 and stabilizes it in panc-1 CSCs after gemcitabine treatment

**Fig 4:**
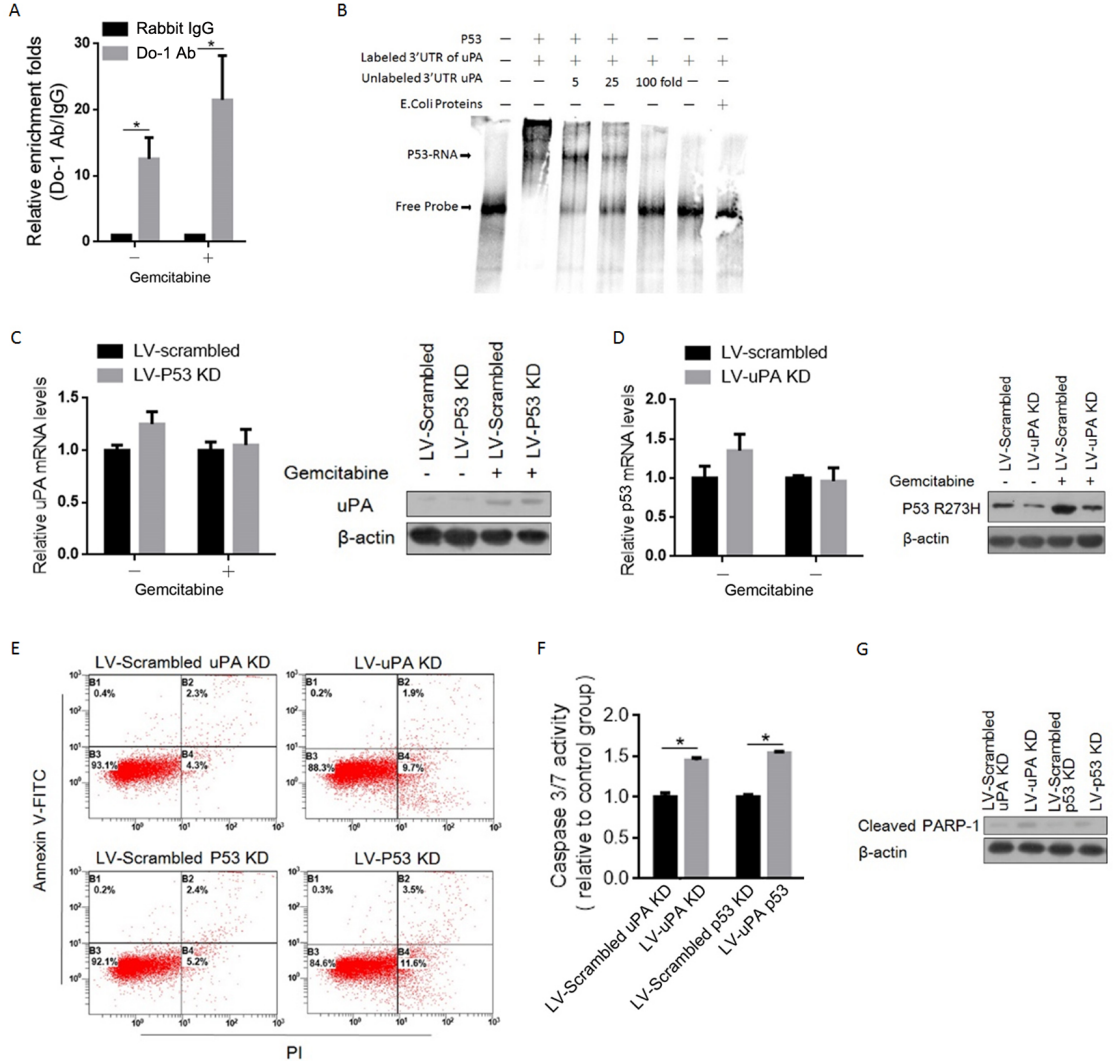
uPA induced by Gemcitabine binds specifically to P53-R273H and partially promoted mitochondrial-dependent apoptosis. (A) RNA-IP assay was performed to confirm the binding of uPA mRNA 3’UTR to P53-R273H in Panc-1 CSCs. (B) EMSA assay was employed for confirming the direct and specific interaction between P53-R273H and fragment of uPA mRNA 3’UTR. (C) The mRNA (left panel) and protein (right panel) levels of uPA after p53 knockdown were measured. (D) The mRNA (left panel) and protein (right panel) levels of P53-R273H after uPA knockdown were measured. (E) The apoptotic condition was detected by flow cytometry using Annexin V-FITC/PI staining, after uPA or P53 knockdown. Caspase 3/7 activity (F) and cleaved PARP-1 (G) were analyzed for determine whether apoptosis induced is dependent on mitochondria. *P<0.05.

Wild type p53 binds to regulatory elements in the 3’UTR of uPA through its C-terminal region (364–393 aa), consequently preventing p53-dependent inhibition of uPA expression [33]. The presence of a pathogenic missense variant in an exon 8 hotspot codon (p.R273H; rs28934576) in panc-1 cells. Raises the question of whether mutant p53 (p53-R273H) interacts with the 3’UTR of uPA. To investigate p53-R273H interaction with the uPA mRNA, we performed RNA immunoprecipitation (RIP)-qPCR using an anti-p53 Do-1 antibody. As shown in Fig 4A, among the immunoprecipitated products, a fragment of the 3’UTR of the uPA mRNA was detected in both gemcitabine treated and untreated panc-1 CSCs. We further examined the binding of a recombinant p53-R273H protein to the 3’UTR of uPA mRNA [33] via electrophoretic mobility shift assay (EMSA). As shown in Fig 4B, the p53-R273H protein formed a specific complex with a Dig-labeled probe, and this complex was abolished by adding a 5 to 100-fold molar excess of unlabeled probe. We then investigated the post-transcriptional role of p53-R273H on the level of uPA protein. As expected, introduction of shP53 into gemcitabine-treated panc-1 CSCs increased the uPA protein level without disrupting the mRNA level (Fig 4C). Surprisingly, the uPA protein post-transcriptionally upregulated the p53-R273H protein level post-transcriptionally, which indicates the potential stabilizing role of uPA on the p53-R273H protein (Fig 4D).

Given the known results of the expression of p53-R273H on inhibiting mitochondria-dependent apoptosis [34], we next investigated whether gemcitabine-induced uPA inhibits apoptosis by stabilizing p53-R273H. The IC_50_ concentration of gemcitabine was employed. As shown in Fig 4E, knockdown of either uPA or p53-R273H promoted gemcitabine-induced apoptosis. Caspases 3 and 7 are activated in the final stages of apoptosis and cleave PARP. As expected, consistent with Annexin-V/PI staining results, knockdown of either uPA or p53-R273H detectably elevated the activities of caspases 3 and 7 (Fig 4F). The cleaved PARP-1 level, a marker of late apoptosis, was increased accordingly (Fig 4G).

### uPA expression attenuates the reactivating effect of CP-31398 on mutant p53-R273H

**Fig 5:**
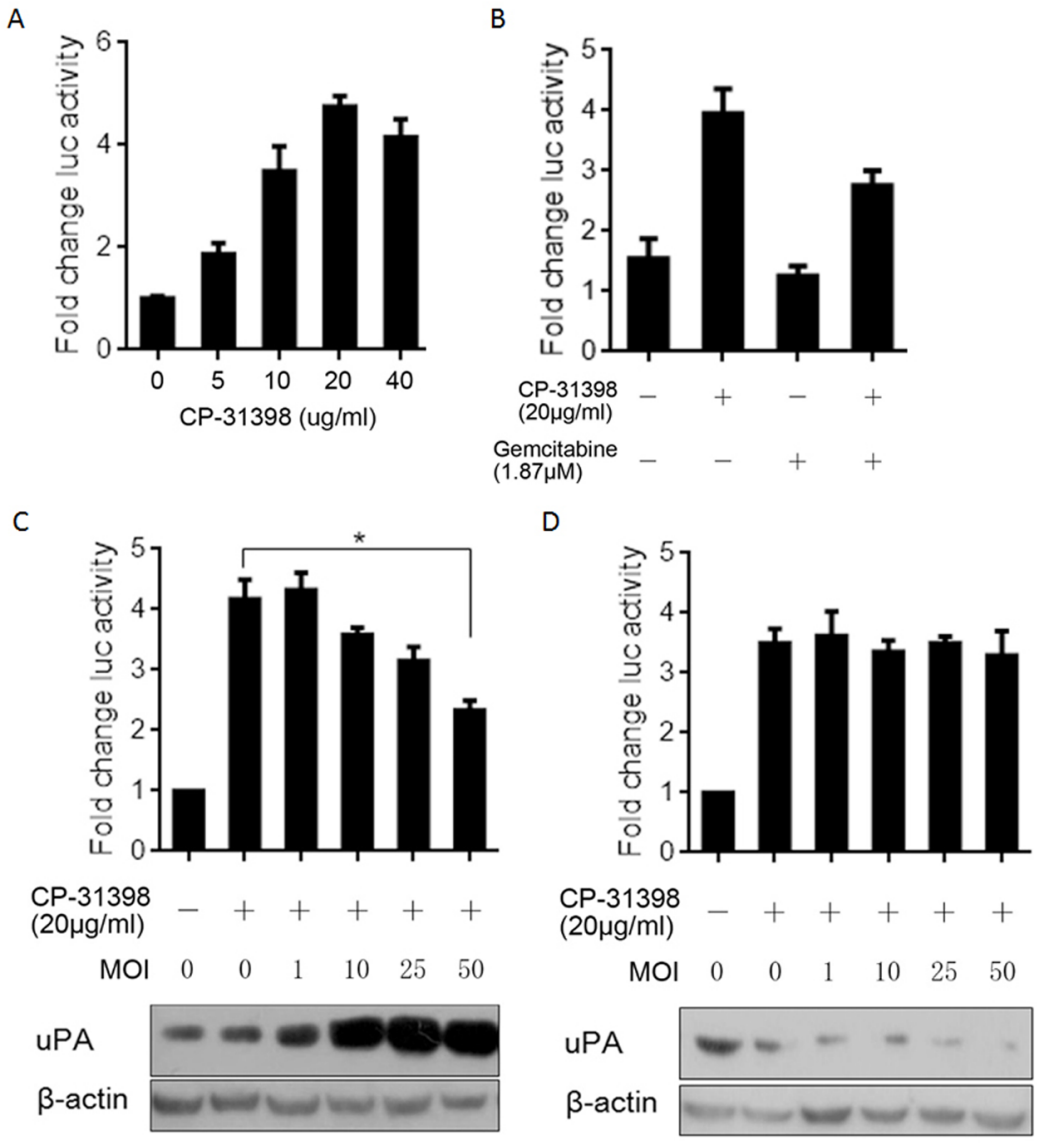
uPA expression attenuates the restorating effect of CP-31398 on mutant P53-R273H. (A) Confirmation of the restorating effect of 5, 10, 20, and 40 ug/ml CP-31398 on transcriptional activity of mutant p53-R273H. (B) Gemcitabine treatment abolished the restoration of CP-31398 on mutant P53-R273H. Upregulated uPA by LV-uPA introduction (C) or downregulated uPA by LV-uPA KD (D) at different MOI affected the transcriptional activity of mutant P53-R273H restored by CP-31398 treatment. *P<0.05.

CP-31398 presents reactivating effects on mutant p53, including p53-R273H [19], and based on this, novel strategies targeting oncogenic mutant p53 have been developed to restore its wild-type conformation and transcriptional activity. We thus addressed whether uPA expression affects restoration of wild-type p53 transcriptional activity of p53-R273H when CP-31398, which is effective for cancers carrying mutant p53, is applied. We first confirmed whether CP-31398 can restore the transcriptional activity of mutant p53. We transiently transfected of panc-1 CSCs with pG13-luc followed by incubation with 0–40 μg/ml CP-31398 for 24 h and then measured luciferase activity using a dual luciferase assay system. As shown in Fig 5A, CP-31398 treatment enhanced the transcriptional activity of mutant p53 compared to the background signal of luciferase. To determine whether gemcitabine treatment modulates the transcriptional activity of mutant p53 that has been restored by CP-31398, panc-1 CSCs transiently transfected with pG13-luc were co-incubated with CP-31398. The data show that gemcitabine treatment detectably inhibited the CP-31398-restored transcriptional activity of mutant p53 (Fig 5B). To determine whether uPA expression is directly involved the regulation of p53’s transcriptional activity, LV-uPA KD and LV-uPA were co-transfected into panc-1 CSCs with CP-31398 treatment. As shown in Fig 5C, the transcriptional activity of mutant p53 was nearly abolished at a high multiplicity of infection (MOI), i.e., 50. However, LV-uPA KD failed to significantly affect luciferase activity and possibly resulted in a low endogenous level of uPA protein (Fig 5D). Taken together, uPA expression disturbed the restoration of mutant p53 due to CP-31398 treatment. However, the exact mechanism remains unknown.

## Discussion

Diagnosis of PDAC is frequently delayed, resulting in a lack of effective treatment options because of its high mortality rate. Gemcitabine is currently widely applied to delay the development of recurrent carcinoma [35], but as monotherapy, it only extends survival by 5 weeks [36]. Three major barriers contribute to the increase in gemcitabine chemoresistance: anatomical, pathophysiological and pharmacological barriers. Despite numerous investigations to date, strategies for overcoming chemoresistance have been largely unsuccessful [37].

Accumulating evidence reveals that CSCs are not only capable of initiating tumours but are also responsible for chemoresistance via several well-known mechanisms [38–40]. In hepatocellular carcinoma (HCC), the portion of CSCs that retain a similar “stemness” feature can partially be attributed to HCC heterogeneity, HCC metastasis/recurrence and chemoresistance [41]. In breast cancer, enriched CSCs contribute to the development of chemoresistance and thus promote metastasis and recurrence [42]. Liu and colleagues found that CSCs in glioblastoma, which present CD133-positive cell surface markers, display significant resistance to conventional chemotherapeutic agents [43]. Accordingly, CSCs constitute a potential target in chemotherapeutic treatment strategies. The goal of our study was to better understand the molecular basis underlying the gemcitabine resistance of pancreatic CSCs, and we demonstrated that CSCs enriched from panc-1 cells present gemcitabine resistance.

ER stress and UPR are upregulated in many cancers, thus presenting great possibility due to their association with drug resistance [44]. Upon ER stress, the branches of UPR balance pro-apoptosis and pro-survival signalling, and activation of IRE1 or PERK can cause a shift towards one of these branches [24]. Our results demonstrate that gemcitabine treatment inactivates the IRE1 branch and activates the PERK branch, indicating a shift from death to survival in panc-1 CSCs. By measuring the ratio of XPB-1/S to XPB-1, it was confirmed that inactivation of IRE1 by gemcitabine treatment of panc-1 CSCs reduces the amount of spliced XBP-1 (XBP-1/S). ATF6, one of three transmembrane proteins within the ER membrane that plays crucial regulatory roles in ER stress [45, 46], also occurs in a spliced form, p50ATF6α, and gemcitabine treatment promoted splicing and translocation to the nucleus. It has been reported that p50ATF6α exhibits a transactivating property after gemcitabine treatment in IRE- and PERK-independent manners, especially towards its downstream target gene GRP78 [47].

GRP78 is a molecular chaperone localized in the ER that has critical roles in protein folding, degradation of misfolded proteins and ER Ca^2+^ binding [48]. GRP78 is positively associated with metastasis of many types of cancers [49–51], and high levels of GRP78 contribute to acquisition of phenotypic cancer hallmarks [52]. As one of the most important hallmarks of ER stress, GRP78 is thought to regulate the phenotypes of cancers in an unknown manner. In this study, we found that ER stress-induced GRP78 upregulated uPA, indicating that uPA might be the downstream target that results in tumour malignancy, including proliferation, migration, invasion and colony formation, because uPA upregulation is tightly correlated with the poor prognosis of pancreatic cancer [22]. Moreover, this hypothesis is supported by the finding that uPA silencing significantly desensitizes panc-1 CSCs to gemcitabine (Fig 3G).

Over the last two decades, numerous reports have described RNA binding by p53 through its C-terminus, though the potential physiological roles of p53-RNA interactions remain unresolved [53]. UPA mRNA is one of these RNAs reportedly bound specifically by p53. Yashodhar and colleagues found that without interfering with its DNA binding activity, p53 binds to a 35-nucleotide sequence within the 3’UTR of uPA [54]. Under the assumption that panc-1 and panc-1 CSCs are homozygous mutant p53 cell lines harbouring hotspot mutations in codon 273 (mutp53-R273H) [55], we first proved that mutp53-R273H binds to the uPA mRNA 3’UTR both in vitro and in cells (Fig 4A & 4B). We failed to detect a disturbance of upregulated uPA on p53’s DNA binding activity because mutp53-R273H exhibits no such specific DNA binding (data not shown) [56]. Surprisingly, knockdown of mutp53-R273H in panc-1 CSCs post-transcriptionally increased the level of p53 protein without altering its mRNA level by inhibiting p53 ubiquitination, indicating the stabilizing effect of the uPA mRNA-mutp53-R273H interaction (Fig 4C & 4D).

In tumours harbouring mutant p53, the strategy for blocking cancer growth or promoting apoptosis using functional p53 has proved to be ineffective [57]. CP-31398, a styrylquinazoline, is able to restore a wild-type-associated epitope on the DNA binding domain of mutant p53, including mutp53-R273H [58]. In human epidermoid carcinoma A431 cells, which express mutp53-R273H, CP-31398 treatment induces p53 downstream target genes, including p21, mdm2, and Bax, in these cells [59]. Thus, evaluated whether CP-31398 induces p53’s transactivating capacity through a luciferase reporter assay and demonstrated that both gemcitabine treatment and increasing amounts of uPA via lentiviral transfection inhibited CP-31398 ability to restore wild-type p53 transactivation (Fig 5). This indicates that gemcitabine-induced uPA might lead to failure of CP-31398 with regard to sensitizing tumour cells to gemcitabine.

In conclusion, we revealed that gemcitabine treatment induces uPA expression by activating ER stress in panc-1 CSCs and that upregulated uPA potentially inhibits CP-31398, which sensitizes panc-1 and panc-1 CSCs by targeting mutp53-R273H. Thus, an improved understanding of the effects of uPA on p53’s DNA binding activity will increase our knowledge of the induction of chemoresistance in pancreatic CSCs.

## Supporting information

S1 Fig uPA knockdown efficiency using LV-uPA KD. Relative mRNA level (A) and protein level (B) of uPA after knockdown by using lentivirus.
(DOCX)

S2 Fig GRP78 knockdown efficiency using LV-GRP78 KD. Relative mRNA level (A) and protein level (B) of GRP78 after knockdown by using lentivirus.
(DOCX)
