# Supplementary material for: Gemcitabine treatment induces endoplasmic reticular (ER) stress and subsequently upregulates urokinase plasminogen activator (uPA) to block mitochondrial-dependent apoptosis in Panc-1 cancer stem-like cells (CSCs)
Source: PLoS One. 2017 Aug 30;12(8):e0184110. doi: 10.1371/journal.pone.0184110 (PMC5576696; doi:10.1371/journal.pone.0184110)
Supplement: S1 Fig — Relative mRNA level (A) and protein level (B) of uPA after knockdown by using lentivirus. (DOCX) [file pone.0184110.s001.docx]

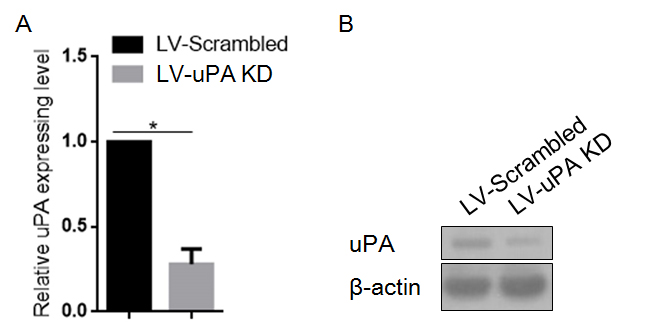
S1 Fig. uPA knockdown efficiency using LV-uPA KD. Relative mRNA level (A) and protein level (B) of uPA after knockdown by using lentivirus
